# Robotic Assembly of Timber Structures in a Human-Robot Collaboration Setup

**DOI:** 10.3389/frobt.2021.768038

**Published:** 2022-01-27

**Authors:** Aljaz Kramberger, Anja Kunic, Iñigo Iturrate, Christoffer Sloth, Roberto Naboni, Christian Schlette

**Affiliations:** ^1^ The Maersk McKinney Moller Institute, SDU Robotics, University of Southern Denmark, Odense, Denmark; ^2^ CREATE Section for Civil and Architectural Engineering, University of Southern Denmark, Odense, Denmark

**Keywords:** learning by demonstration, assembly of timber structures, digital twin, robotic assembly, robotic fabrication

## Abstract

The construction sector is investigating wood as a highly sustainable material for fabrication of architectural elements. Several researchers in the field of construction are currently designing novel timber structures as well as novel solutions for fabricating such structures, i.e. robot technologies which allow for automation of a domain dominated by skilled craftsman. In this paper, we present a framework for closing the loop between the design and robotic assembly of timber structures. On one hand, we illustrate an extended automation process that incorporates learning by demonstration to learn and execute a complex assembly of an interlocking wooden joint. On the other hand, we describe a design case study that builds upon the specificity of this process, to achieve new designs of construction elements, which were previously only possible to be assembled by skilled craftsmen. The paper provides an overview of a process with different levels of focus, from the integration of a digital twin to timber joint design and the robotic assembly execution, to the development of a flexible robotic setup and novel assembly procedures for dealing with the complexity of the designed timber joints. We discuss synergistic results on both robotic and construction design innovation, with an outlook on future developments.

## 1 Introduction

In recent years, the field of construction robotics has gained in popularity, primarily because of a shifting mindset towards a sustainable, reusable and carbon-efficient society. One of the leading construction methods in this domain is robotic timber construction which focuses on sustainable design, manufacturing and assembly processes. The widespread diffusion of computational workflows to link design and simulation to real-world assembly operations has opened opportunities for efficient non-standard timber construction and increased the overall level of automation. With the progressive diffusion of robotic and automation technologies, the construction domain can also harness a higher level of automation in their production processes, essential to meet increasing demands for material and carbon efficiency and relieving labor-intensive and repetitive tasks. While the robotic fabrication of timber structures with Computer Numerical Control (CNC) technology relies on consolidated methods (Yuan et al., 2016), the automated assembly of wood structures is still a challenging task, especially in development of highly efficient structures that require custom design and assembly strategies. Working with a non-homogeneous material e.g., wood presents various challenges for robotic-driven assembly processes. Piece-specific material features, weather-related shape variations, geometrical imprecisions and occurring tolerances shall be carefully considered in the overall process. This paper presents a new framework for tackling such challenges in the context of the robot-based collaborative assembly of timber truss structures connected through novel lap-joint connections that interlock elements through complex assembly motions. These elements guarantee mechanical capacity through tight insertions that, however, can be difficult to achieve with common industrial robots and assembly strategies. Therefore, we introduce the concept of Learning by Demonstration (LbD) strategies based on Dynamic Movement Primitives (DMP) to teach robots how to perform such operations, thus omitting conventional robot programming, which for task like this is very complex. The first part of the paper introduces a robot-friendly design case study for a structural truss based on discrete wood elements, presenting innovative joinery features that allow assembly and disassembly through complex assembly motions. The second part describes the underlying digital twin and the pipeline connecting the phases of design, task planning and assembly. Lastly, adaptation procedures based on the task dynamics which arise during the assembly execution are presented. The effectiveness of the framework will be shown on a timber structure assembly executed by two collaborative robots.

The main contributions of the paper are

• a digital framework, linking the design and simulation of timber trusses with the robot based assembly procedures through a digital twin,
• design of novel interlocking timber truss joints, taking into account the capabilities and limitations of a collaborative robot,
• robotic LbD assembly method with adaptation capabilities, for execution of the demonstrated motion under various workspace conditions.

## 2 State of the Art

Extensive research has been conducted internationally with a particular effort on enhancing the robotic assembly of timber structures (Stumm et al., 2018; Thoma et al., 2019; Helm et al., 2016; Eversmann, 2017; Søndergaard et al., 2016; Naboni et al., 2021; Kunic et al., 2021b). Recent works have been looking into robotic assembly strategies for structures that rely on friction-based wood/wood connections to achieve structural strength through tight-fitting. While these techniques are found in historical traditional construction methods worldwide (Zwerger, 2015), they have been poorly implemented in current construction practices in favor of more straightforward techniques where steel plates are added to fixate the wooden elements (Leśko, 2016). The process is materially inefficient and presents strong geometrical limitations that compromise the effective construction possibilities. Advancements in CNC technologies and automation give new possibilities for fast and efficient fabrication of traditionally established wood-wood joinery, which used to be time consuming and required a high level of dexterity from the woodworking craftsmen. Experimental structures have been constructed with the use of interlocking half-lap joints to connect straight timber beams (Chai et al., 2019); integrative finger joints for the construction of plate-based (Krieg et al., 2015) and folded (Robeller et al., 2017) structural shells; interlocking timber tiles shaped like a puzzle, held by tension and gravity, to form structural arches (Kuma and Associates, 2020). The assembly of these structures primarily relies on manual work, as the tight interlocking of such connections requires complex robot tool path planning and advanced force control to be realized.

In the existing literature, we can find three main approaches to the problem of tight fit wood assembly. The first one relies on force/torque monitoring and poses data collection as seen in (Stumm et al., 2018; Stumm and Brell-Çokcan, 2019) and in the case of wood/wood lap joints assembly for spatial frame structures thought reinforcement learning methods (Apolinarska et al., 2021). The second method relies on computer vision to provide the necessary localization of the wooden elements and validation of the assembly operations, combined with geometrical adaptations of joinery elements to gain positioning precision during the assembly of reversible discrete structures (Kunic et al., 2021a).

Similarly, plate structures with multiple through-tenon joints have been automatically assembled, relying on fiducial markers and chamfered geometries that guide linear assembly motions (Rogeau et al., 2020). The third method is based on custom mechanical devices to apply additional clamping forces in the assembly process. Leung et al. (2021) utilizes custom-built remote-controlled clamps to apply large assembly forces and correct misalignment’s between wooden elements. At the same time Robeller et al. (2017) introduced a custom-built end-effector for vibration-assisted assembly of through-tenons joints, where the assembly forces are monitored with a load cell. Koerner-Al-Rawi et al. (2020) utilized two coordinated industrial robotic arms to assemble wood lattices through rotation and locking, building upon traditional Japanese Chidori joints.

In the work mentioned before, conventional industrial robots were utilized in order to automate the timber assembly tasks. In recent years we can see a shifting trend where collaborative robots are being utilized for collaborative tasks (Kunic et al., 2021a) in timber construction, where the workspace is shared between the human and the robot. Those systems follow the idea of the Industry 4.0 (Bragança et al., 2019), where the industrial systems are linked with the cloud, are able to be programmed with service/skill based programming structures (Schou et al., 2018) and enable interaction with agents, such as digital twins in a working environment (Malik and Brem, 2021). Furthermore, new manufacturing trends, focusing on efficiency, user friendliness and flexibility of the manufacturing process were introduced (Gašpar et al., 2020), replacing the need for specialized automation equipment, which governed the production processes in the past. Furthermore, the newly introduced automation technologies paved the way for methods such as LbD (Billard et al., 2008) and interaction control with the environment, thanks to the integrated sensors and controllers of the automation systems.

For a standard LbDscenario, robot trajectories describing the task are recorded (Deniša and Ude, 2015) by a human operator. While this information is adequate for robot tasks where the robot is not in contact with the human or the environment, for in-contact tasks e.g., assembly (Abu-Dakka et al., 2015) or polishing (Gams et al., 2013), dynamic data arising during the demonstration and execution has to be considered.

In order to execute tasks in contact with the environment, the execution framework has to take into account also the adaptability and generalizability aspect of the robot motion. Therefore, in many publications, demonstrated robot data is represented with Dynamic Movement Primitives (Ijspeert et al., 2013; Ude et al., 2014). With this framework, kinematic and dynamic robot trajectories and sensor readings can be represented in unified way, and can easily be adapted to a new situation with the help of modulation and time scaling. Furthermore, the robot data can be reused for synthesizing new movements (Kramberger et al., 2017; Lončarević et al., 2021) from a database of pre-recorded movements.

For in-contact tasks, where uncertainties e.g., manufacturing tolerances and material inconsistency, play a major factor, the before mentioned framework can efficiently be exploited, when coupled with a force control strategy as shown in Rozo et al. (2013), Koropouli et al. (2012), and Kormushev et al. (2011). Moreover, adaptation can also be achieved by changing the impedance of the robot, depending on the requirements of the task. By changing the stiffness parameters of the impedance controller, the interaction forces can be adjusted between the robot and the environment (Buchli et al., 2011). Pastor et al. (2011) introduced a method for real-time adaptation of the demonstrated robot motion depending on the measured force/torque data. The authors present an adaptive controller for learning and adaptation of demonstrated motion, where measured and learned force/torque feedback was utilized for in-contact robot tasks. Furthermore, a review of online adaptation of impedance control parameters for human robot interaction (Müller et al., 2018) and in-contact execution tasks are presented in the work of Abu-Dakka and Saveriano (2020).

## 3 Case - Study of Digital Design and Assembly of Complex Timber Truss Structures

**FIGURE 1:**
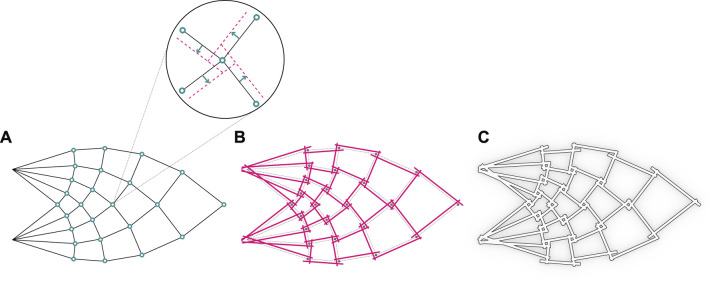
The process of structural design inspired by Michell’s optimal truss: **(A)** Michell’s optimal truss, **(B)** Revised structure with reciprocal connections, **(C)** The final structure made of timber studs.

The urge to build more sustainable and circularity oriented in combination with modern computational design tools opens new perspectives for the construction industry. Driven by such motivations, we introduce a concept for reciprocal timber truss structures featuring innovative cross-lap interlocking joints. The presented design method promotes material and structural efficiency while proposing robot-friendly and assembly-aware features. The global structural design method is inspired by Antony Michell’s famed theoretical study (Michell, 1904) on the optimal truss structures that use a minimal amount of material while providing maximum structural capacity. In particular, we focus on the case of a cantilevered element with a vertical point load at the full span length. A frame based on quad elements is generated following ideal load paths (Figure 1).

Our approach revisits such a structural solution, shifting from a typical strut-and-pin system to reciprocal connections (Larsen, 2007) based on interlocking cross-lap joints. This method enables timber-timber joinery without the need for additional steel fasteners while still providing structural integrity.

**FIGURE 2:**
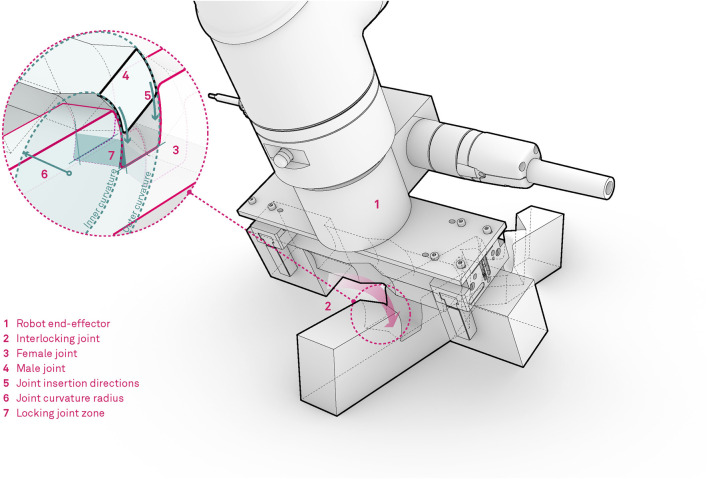
Visual description of the truss design for robotic assembly.

The cross-lap joint is here re-invented and designed with three-dimensional interlocking features derived from an idealized assembly trajectory that locks into position two or more wood studs through spatial translation and rotation. An initial inclined angle is followed by a rotational motion as shown in Figure 2, leaving no margin for the studs to be moved or removed unless the reversed trajectory is applied. The filleted edges and semi-circular cross-section of the joint ensure a smooth robotic positioning and a tight interlocking in the joint. The wood studs are placed along the compression and tension elements from Michell’s truss. To manufacture such a joint, CNC milling has to be adopted in order to ensure the described properties.

## 4 Methodology

In this section, the relevant methods outlining the digital robotic timber assembly framework, will be presented.

### 4.1 Digital Design Framework for Robot-Based Assembly

In this work computational design algorithms are developed to manage the global and local design parameters and transform these into robot targets and plan assembly trajectories. A bi-directional communication between the computer design environment and the physical setup is established, and constant updates on the overall assembly process and specific design targets are possible due to feedback from the construction procedures. The process is further aided by the human demonstration method, which allows for contact rich assembly skills to be transferred to the robot. Following Cimino et al. (Cimino et al., 2019), we apply digital twins to digitally mirror the state of the physical entities (Kritzinger et al., 2018) (i.e. timber structures and robot technologies) in order to support the analysis, process planning and operation monitoring of the setup.

**FIGURE 3:**
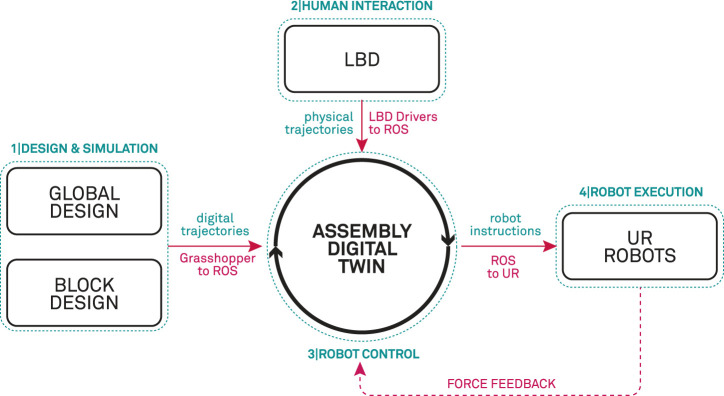
Outline of the digital framework, linking the design and simulation, LbD and the physical level of the robot execution in an assembly digital twin.

The overall digital twin robotic assembly framework (outlined in Figure 3) is organized in the following phases:

• In *phase* 1 – Design & Simulation of wooden blocks is implemented in Grasshopper (Rutten and McNeel, 2007) which represents a visual algorithmic editor plugin for Rhinoceros^1^, commonly used in the architectural design industry. In this stage the 3D model of the global truss structure is designed and stored. Additionally, the global structure contains the information of how each specific 3D modeled timber block is utilized in the global structure. This data is directly linked to a parametric structural model that can autonomously generate such data for different design cases. For more information on the parametric design of timber structures we direct the reader to our previous work Naboni et al. (2021). On this basis, geometric data is utilized to plan and simulate robot trajectories from the material stock feed to the location of insertion above a specific joint connection.
• In phase 2 – Human demonstration of the assembly task is recorded and interpreted. The demonstrated data represents a coordinated robot movement by physically grabbing and guiding the robot along the desired path, consisting of translational and rotational trajectories represented in operational space.
• phase 3 – Robot Control works as a digital twin with Robot Operating System (ROS) as the underlying communication between the service oriented control structures. Each robot action e.g., point to point movement, screwdriver operation, etc., is represented as service with specific inputs and outputs Naboni et al. (2021). Section 5.3 describes how the demonstrated data is utilized in the service oriented control structure. In this phase, all of the collected digital planning information from phase 1 and physical trajectories from phase 2 are compiled to a set of composite instructions giving an assembly sequence for a specific timber structure.
• In phase 4 – Physical robots are used to execute the tasks following the sequence of operations (from phase 3). In this work we make use of the collaborative robots with integrated force/torque sensors, by which the data measured during the assembly execution, is collected and sent back to the digital twin as a form of feedback – which is used to evaluate the success rate of a specific assembly execution (see Sec. 5.2). If the execution is successful the assembly digital twin schedules the next task.

### 4.2 System Description and Robot Cell Design

**FIGURE 4:**
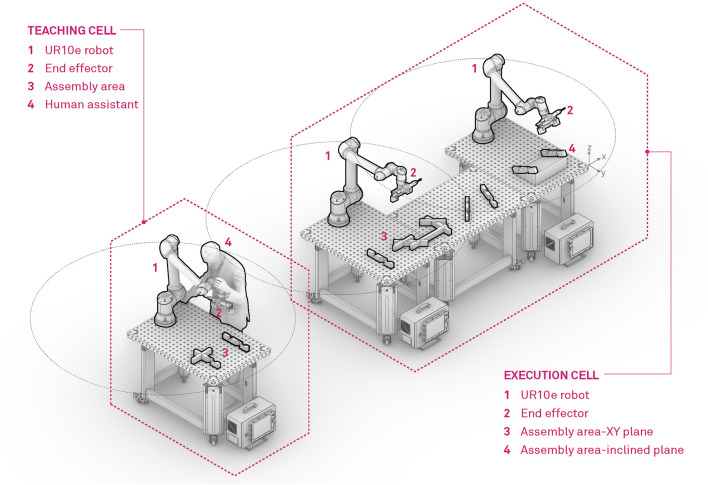
Robot concept cell, designed for fabrication of timber structures. On the left hand side of the figure, the teaching cell is represented, where the operator teaches the robot actions with the help of LbD. The right hand side, represents the execution cell, where the thought actions with a combination of manipulation and robot screwing actions are deployed for robotic assembly of timber structures.

The experimental set-up for the robotic assembly of complex timber truss structures is based on two separate robotic cells, namely the *Teaching cell* and the *Execution cell* which are outlined in Figure 4. Both cells are equipped with universal Robot UR10e robots mounted on a Siegmund welding table. The *Teaching cell* is primarily designed for human-robot collaboration where LbD methods are applied to teach the robot highly complex and articulated moves which are required for assembly of tight interlocking timber joints. On the other hand, the *Execution cell* consists of two robots with dedicated tools, mounted on three tables, covering a full range of 4.6 m. With this configuration large timber structures can be assembled. Furthermore, in the execution cell, we combine timber layered assembly methods, consisting of pick and place, automatic and collaborative robotic screwing and vision localization of timber trusses, presented in our previous work Naboni et al. (2021), with the newly developed methods based on LbD.

**FIGURE 5:**
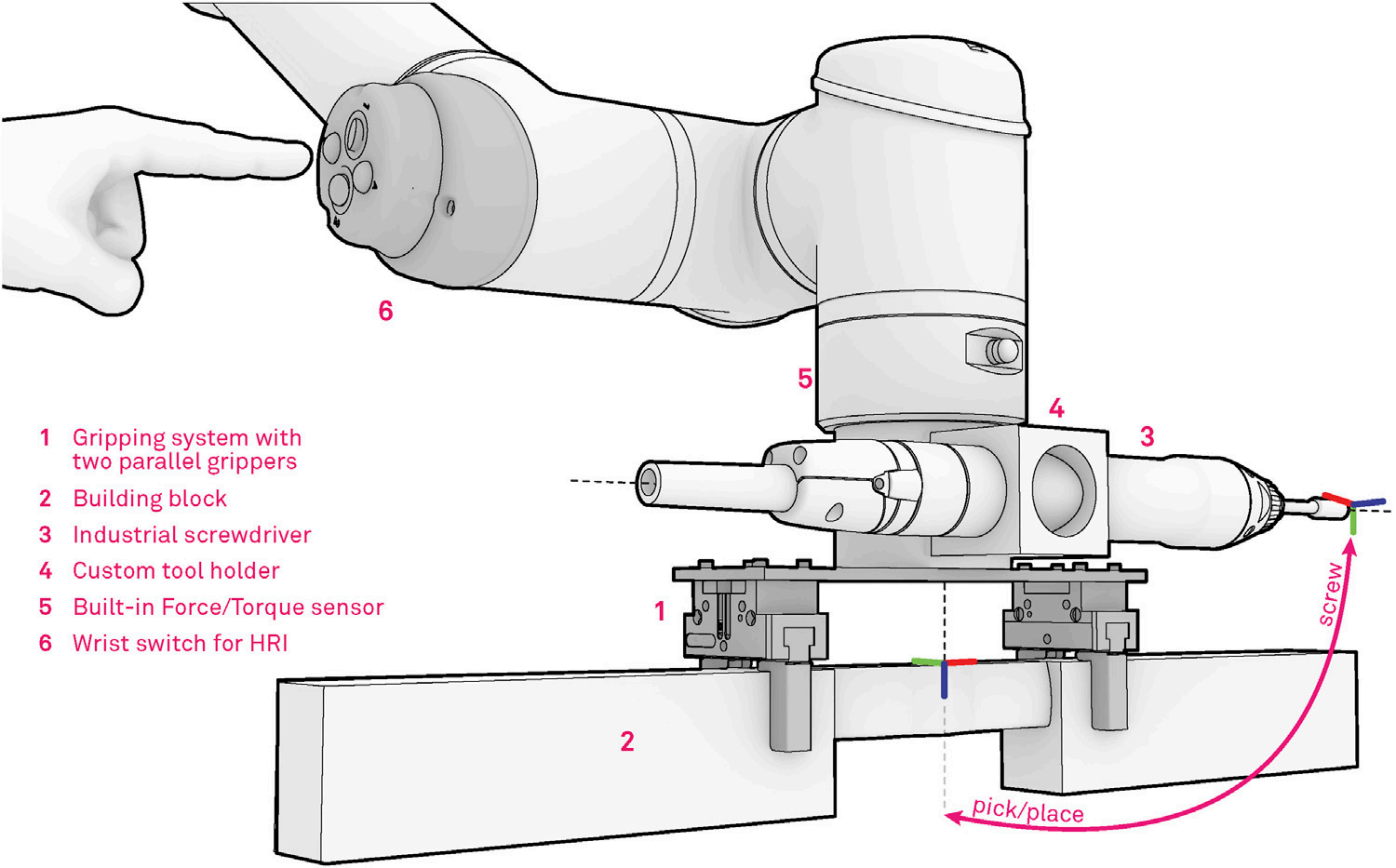
Robotic tool for timber assembly, consisting of two parallel industrial pneumatic grippers, automatic screwdriver and the HRI interface.

Each robot is equipped with a custom designed end-effector suitable for handling of wooden trusses and assembly of complex and tight timber joints, which allows for both the manipulation and screwing procedures (Figure 5). The gripper tool consists of two parallel Schunk JGP-64 pneumatic grippers which are rigidly mounted on a mounting plate at a distance of 20 cm. The mounting distance is designed based on the wooden stud design and allows for handling studs from 0.25 to 1.5 m. For this purpose specialized gripper fingers were designed and manufactured from sheet metal, featuring bend metal contact features which are suitable for grasping timber studs and can accommodate eventual manufacturing and material imprecisions.

Additionally, an industrial screwdriver is mounted within a custom-made steel flange, placed at a 90-degree angle from the gripper to avoid any form of collision between the tools while enabling multi-phase assembly procedures. The developed assembly system also takes full advantage of the built-in force-torque sensor which is placed within the robot flange and is used to record forces and torques during assembly procedures. The information is then passed to the digital twin for verification purposes or directly to the robot controller for adaptation purposes during the assembly. Finally, a custom interface for Human Robot Interaction (HRI), is mounted on the fourth joint of the robot. In our previous work (Naboni et al. (2021)), we applied this interface for collaborative task executions with a human operator. In this work we make use of the interface primarily in the demonstration phase. The human demonstrator can trigger various modes e.g., robot free drive, start/stop recording, gripper actions, while demonstrating the desired movement. With this setup the operator can easily demonstrate the assembly knowledge, which can later be executed in an assembly sequence.

## 5 Human – Robot Collaboration Setup for Timber Assembly

For LbD in connection with assembly tasks, several methods commonly known in robotics (Billard et al., 2008) can be employed. LbD and reuse of robot motion represents an easy, efficient and user-friendly way to program complex robot motion, not only for assembly but also for other tasks in robotics e.g., robotic reaching, polishing and grinding.

In construction automation, more specifically construction of timber structures, two main robotic approaches are arising from the robotic community. The first deals with layered design, where the robot paths can be extracted from the design directly and the designed timber blocks are assembled in pick and place type fashion. The second approach deals with more complex assembly structures, where the joints of the structures are designed in a way that they are self locking. In this case, the human operator has to intervene and execute the assembly (Naboni et al. (2021)), because programming of robot trajectories is very cumbersome and it takes a lot of resources and time. Some recent work in this field proposes reinforcement learning (Apolinarska et al. (2021)), to learn a specific assembly policy in simulation and then transfer the knowledge to a robotic system. This approach gives a policy which can be used for a single assembly setup and is not easily generalizable. To overcome this hurdle, in this paper we make use of LbD for leaning assembly policies for complex joints in timber assembly, which can be easily executed, adapted and generalized to various timber structure constraints.

### 5.1 Demonstration of Assembly Policies for Timber Structures

**FIGURE 6:**
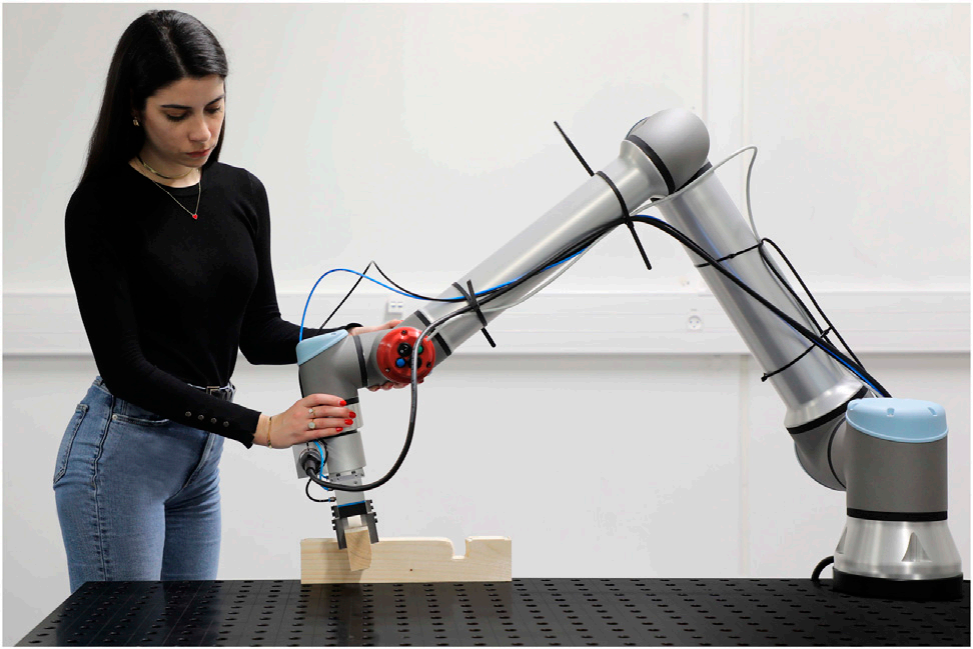
Demonstration of the robot based assembly policy for timber structures.

In this work we exploit one of the LbD methods called kinesthetic guiding (Hersch et al. (2008); Kramberger et al. (2017)) shown in Figure 6, as the main method for demonstrating human skills. Kinesthetic guiding enables us to record robot data by simply grabbing the robot and guiding it until the desired motion is achieved. In the process we record the 6-D Cartesian space and joint space movement trajectories. Please note that this method cannot be exploited on all robot manipulators, the manipulator must have the capability of active gravity compensation e.g., free drive, which is a common feature in collaborative robotics. For industrial robots, where this feature is commonly not available, motion demonstration with haptic devices can be applied.

In order to record the desired data, a human operator physically guides the robot along the desired trajectory and thus receives the same feedback from the environment as the robot. Consequently, the demonstrated trajectories are from the human perspective optimal, because the human can transfer the desired motion and adapt the demonstration to account for the environmental discrepancies if needed. The actual data we record with every demonstration is the following:

$$D_{d}={\left\{p_{i},q_{i},\varsigma_{i},t_{i}\right\}}_{i=1}^{T},$$ (1)

where **p**_*i*_, **q**_*i*_ are the measured Cartesian space positions and orientations (represented as unit quaternions) and ***ς***_*i*_ are the joint angles measured at every time step during the demonstration *t*_*i*_, *i* = 1, … , *T* and *T* denotes the number of measurements in the training set. For a more detailed explanation of orientations encoded in the quaternion space we refer the reader to the following papers Ude et al. (2010, 2014). In addition to the kinematic data, we also record the corresponding dynamic characteristics of the demonstrated motion

$$F_{d}={\left\{F_{m},M_{m},t_{i}\right\}}_{i=1}^{T},$$ (2)

**F**_*m*_ represents the recorded forces and **M**_*m*_ the recorded moments with an integrated force-torque sensor, during the replay of the demonstrated assembly task where the recorded joint space trajectory consisting of a time series of recorded joint angles ***ς*** is re-executed, represented in the base frame of the robot. With this approach, only the relevant dynamic data is recorded and it will be utilized for comparison purposes to outline the effectiveness of the adaptation procedures explained in the next section.

### 5.2 Assembly Policy Representation for Assembly of Timber Struts

In the paper we are primarily focusing on Cartesian space trajectories; therefore, to represent the recorded motion we exploit the Cartesian space Dynamic Movement Primitives (CDMPs) originally presented by Ude et al. (2014). The framework consists of a mass, spring, damper system with well-defined attractor dynamics providing a stable system for trajectory representation (Ijspeert et al. (2013)). CDMPs generate an autonomous control policy, which is robust to external perturbations and can be utilized for modulation of robot trajectories. The second order dynamical system models the progression of the trajectory and can be easily learned from a human demonstration. In CDMPs, the positional part of the demonstrated movement is treated in the same way as in the standard DMP formulation (Ijspeert et al. (2013)), whereas the orientational part of the trajectory is represented by unit quaternions and require special treatment, both in the nonlinear dynamic equations and during the integration. The system is represented with the following set of equations:

$$\begin{array}{ll} \left[\begin{array}{c} \tau \dot{z}\\ \tau \dot{\eta } \end{array}\right] & =\left[\begin{array}{c} \alpha_{p}\left(\beta_{p}\left(g^{p}-p\right)-z\right)\\ \alpha_{q}\left(\beta_{q}2\log\left(g^{q}\bar{q}\right)-\eta \right) \end{array}\right]+\left[\begin{array}{c} f^{p}\left(s\right)\\ f^{q}\left(s\right) \end{array}\right] \end{array}$$ (3)

$$\begin{array}{ll} \left[\begin{array}{c} \tau \dot{p}\\ \tau \dot{q} \end{array}\right] & =\left[\begin{array}{c} z\\ \frac{1}{2}\eta *q \end{array}\right] \end{array}$$ (4)

$$\begin{array}{ll} \tau \dot{s} & =-\alpha_{s}s \end{array}$$ (5)

where *τ* represents the time scaling of the movement, ***g*** ^*p*^, ***g*** ^*q*^ are the desired final positions ***g*** = [*x*, *y*, *z*] and orientations of the demonstrated movement in Carthesian space. In this framework, the orientations are represented as a unit quaternions **q** = *v* + *u* ∈ *S* ^3^, where *S* is a unit sphere, representing a singularity free representation. For additional information on the mathematical representation in quaternion space, we direct the reader to the work of Ude et al. (2014). ***z*** and ***η*** represent the time scaled linear and angular velocity of the dynamical system and **p** and **q** correspond to the position and orientation of the demonstrated motion. The system is initialized at **p** = **p** ^0^, **q** = **q** ^0^, corresponding to the start pose of the demonstrated motion. Furthermore, parameters *α*_*p*_, *α*_*q*_, *β*_*p*_, *β*_*q*_ and *α*_*x*_ are related to the mass-spring-damper control parameters of the second order system, which influence its behavior. If the parameters are set in the following manner; *τ* > 0, [*α*_*p*_, *α*_*q*_] = 4 [*β*_*p*_, *β*_*q*_] > 0 and *α*_*x*_ > 0, then the dynamical system has a unique and stable point attractor at [**p**, **q**] = [***g*** ^*p*^, ***g*** ^*q*^], ***z*** = 0.

Additionally, *s* represents the exponential phase variable, which synchronizes all of the CDMPs in the system. Given the initial condition *s* (0) = 1, Eq. 5 can be solved analytically by *s*(*t*) = exp (*α*_*x*_ *t*/*τ*), which characterizes the end of the encoded movement. Additionally, the shape of the demonstrated movement is encoded as a non-linear forcing term ***f***_*p*_(*s*) and ***f***_*q*_(*s*) for Cartesian positions and orientations separately. The term is a combination of radial basis functions, which essentially enables the robot to follow a demonstrated movement from the beginning to the end of the trajectory.

$$\begin{array}{ll} f_{p}\left(s\right) & =\frac{\sum_{k=1}^{N}\psi_{k}\left(s\right)w_{k}^{p}}{\sum_{k=1}^{N}\psi_{k}\left(s\right)}s, \end{array}$$ (6)

$$\begin{array}{ll} f_{q}\left(s\right) & =\frac{\sum_{k=1}^{N}\psi_{k}\left(s\right)w_{k}^{q}}{\sum_{k=1}^{N}\psi_{k}\left(s\right)}s, \end{array}$$ (7)

$$\begin{array}{ll} \psi_{i}\left(s\right) & =\exp\left(-h_{k}{\left(s-c_{k}\right)}^{2}\right), \end{array}$$ (8)

The afore mentioned free parameters, $w_{k}^{p},w_{k}^{q}\in R^{3}$ respectively represent the positional and orientational part of the demonstrated trajectory. The radial basis functions *ψ*_*k*_ outlined in Eq. 8 can be defined in many ways, in this paper we follow the proposed approach from Ude et al. (2010). More information on how to encode and reconstruct data with CDMPs, can be found in Ude et al. (2014).

### 5.3 Coupling the Demonstrated Data With the Digital Twin

The demonstrated knowledge, explained in the previous section, is represented as a self-contained LbD service in the execution framework. The service-based architecture provides a simple and efficient way to extend and plug in new services to the assembly digital twin. In this section we will explain the idea of the LbD service and how it is incorporated into the assembly digital twin.

The LbD service represents a self-contained framework for execution of demonstrated trajectories encoded with CDMPs. The input to the service is a set of target poses supplied by the global designer, which correspond to the target locations of the wooden blocks in the final assembly. The idea of the LbD service is to incorporate the supplied information and modulate the demonstrated trajectory to prepare it for execution with adaptation at any location in the workspace of the robot.

#### 5.3.1 Modulation of the Demonstrated Motion, Based on the Assembly Digital Twin Input

DMPs and CDMPs are known for their modulation properties (Ijspeert et al. (2013)), this means that if a user changes the target goal of the position or orientation representation, the DMP with its dynamics ensures stable convergence. In order to exploit this property, in this work we adopted the modulation formulation presented in Ginesi et al. (2021) for the positional part of the trajectory and extend it to take into account also the orientational part of the trajectory. Thus, we reformulate the standard CDMP equations (Eq. 3-4), to account for the modulation factor, given by the rotation matrix **R** ^*p*^ for the positional part (Ginesi et al. (2021)) and a novel orientation factor **R** ^*q*^ represented in unit quaternion space.

$$\left[\begin{array}{c} \tau \dot{z}\\ \tau \dot{\eta } \end{array}\right]=\left[\begin{array}{c} \alpha_{p}\left(\beta_{p}\left(g_{new}^{p}-p\right)-z\right)\\ \alpha_{q}\left(\beta_{q}2\log\left(g_{new}^{q}*\bar{q}\right)-\eta \right) \end{array}\right]+\left[\begin{array}{c} R^{p}f^{p}\left(s\right)\\ R^{q}f^{q}\left(s\right) \end{array}\right]+\left[\begin{array}{c} F_{c}\\ M_{c} \end{array}\right]$$ (9)

The multiplication of the forcing term with the modulation factors accounts for the orientational and translational transformation of the entire demonstrated trajectory in accordance to the given inpute pose. Furthermore, we couple the reformulated equations with a force coupling term to enable adaptation of the demonstrated motion, based on the force input during the execution, more will be presented in Section 5.4.

In order to modulate the demonstrated trajectory with the associated target poses, given by the global design unit, we first have to calculate the relative start pose $p_{new}^{0},q_{new}^{0}$ of the demonstrated trajectory, which corresponds to the new goal pose $g_{new}^{p},g_{new}^{q}$ provided by the global design unit. To obtain this information, we calculate the relative transformation between the original and new goal of the associated trajectory for both positions and orientation

$$p_{T}^{p}=g_{new}^{p}-g^{p},$$ (10)

$$q_{T}^{p}=g_{new}^{q}{\bar{g}}^{p}.$$ (11)

In the equations $p_{T}^{p}$ and $q_{T}^{q}$ represent the positional and orientational transformation between the demonstrated positional and orientational goal poses **g** ^*p*^, **g** ^*q*^ and the new target goal poses. The new updated start position can be calculated as $p_{new}^{0}=p_{0}+p_{T}^{p}$ and the updated start orientation $q_{new}^{0}=q_{T}^{q}q_{0}$ , respectively. For the positional part we first find the transformation vector between the new start and goal position of the trajectory $p_{T,new}^{p}=g_{new}^{p}-q_{new}^{0}$ and the transformation vector $p_{T,train}^{p}=g_{train}^{p}-q_{train}^{0}$ , corresponding to the original trained trajectory. The relative transformation angle *γ*_*ang*_ and the corresponding axis ***γ***_*ax*_ are calculated with the following equation

$$\gamma_{ang}=\arccos\left(\frac{p_{T,new}^{p}p_{T,train}^{p}}{\left|p_{T,new}^{p}||p_{T,train}^{p}\right|}\right),$$ (12)

$$\gamma_{ax}=|p_{T,1}^{p}\times p_{T,2}^{p}|.$$ (13)

In order to get the rotation matrix representation **R** ^*p*^, we transform the axis angle representation to rotation matrix as outlined in Siciliano et al. (2008).

Calculating the relative transformation in orientation space represented with unit quaternions needs a special treatment. Unlike positions, which can be represented as a 3-D vector, the orientation quaternions are represented in *S* which is a unit sphere, therefore the transform between two unit quaternions is represented as an angular velocity that rotates the first quaternion e.g., **q**_0,*new*_ into the second quaternion $g_{new}^{q}$ (Ude et al. (2014)) and is calculated with the following equations

$$q_{T1}^{q}=2\log\left(g_{new}^{q}{\bar{q}}_{0,new}\right),$$ (14)

$$q_{T2}^{q}=2\log\left(g_{train}^{q}{\bar{q}}_{0,train}\right).$$ (15)

With this formulation we get two transformation vectors $q_{T1}^{q}$ and $q_{T2}^{q}$ mapping from $S^{3}\to R^{3}$ , which can with the help of Eqs 12, 13 give a rotation angle and axis. To get the rotation matrix representation **R** ^*q*^ used in Eq. 9, we transform the axis angle representation to rotation matrix. With this extension to the normal CDMP formulation we can simply transform the encoded demonstration to any arbitrary location in the workspace of the robot.

### 5.4 Adaptation of the Modulated Demonstrated Motion

In order to ensure a successful execution of the transformed demonstrated motion, we exploit the interaction forces measured during the execution with a force-torque sensor mounted at the wrist of the manipulator. The force measurements are used in a force control setting in order to add compliance to the system. The aim of this approach is to adapt the robot motion to small displacements, arising from the imperfections in the manufacturing process of the timber studs, positioning errors in the workspace and robot tool calibration imprecisions. The problem of unforeseen displacements is that, during the assembly process, small displacements can cause large forces being exerted on the work peace or the manipulator and consequently leading to a failed execution or even damage the hardware. In the presented approach we do not aim to control a specific force during the assembly, but to follow the demonstrated kinematic trajectory in order to execute the complex joint assembly procedure. For this reason we extend the original CDMP formulation (Eq. 3) with applying a compliance PI controller directly to the acceleration level of the CDMP (Eq. 9) enabling adaptation of the encoded motion directly while being executed. The compliance controller outputs are defined for the positional (**F**_*c*_) and orientational (**M**_*c*_) part of the CDMP, separately as follows:

$$F_{c}=K_{p,F}\left(F_{d}-F_{m}\right)+K_{i,F}\int \left(F_{d}-F_{m}\right)dt,$$ (16)

$$M_{c}=K_{p,M}\left(M_{d}-M_{m}\right)+K_{i,M}\int \left(M_{d}-M_{m}\right)dt.$$ (17)

In the equations **F**_*d*_, **M**_*d*_ represent the desired forces and torques and **F**_*m*_, **M**_*m*_ the measured forces and torques, for each tool axis separately. ***K***_*p*,*F*_, ***K***_*p*,*M*_ and ***K***_*i*,*F*_, ***K***_*i*,*M*_ are positive defined gains of the PI controller. With this setup we can efficiently adapt to the environmental displacement, while minimising the interaction forces, ensuring a safe and reliable execution of the assembly task.

## 6 Experiments

In this section we will present the evaluation results of the proposed timber assembly framework. The experiments were conducted on the execution platform described in Sec. 4.2. The evaluation of the assembly approach, was executed several times at arbitrary placement locations of the mock up wooden studs in the workspace of the robot.

### 6.1 Demonstration of the Complex Joint Assembly Task

**FIGURE 7:**
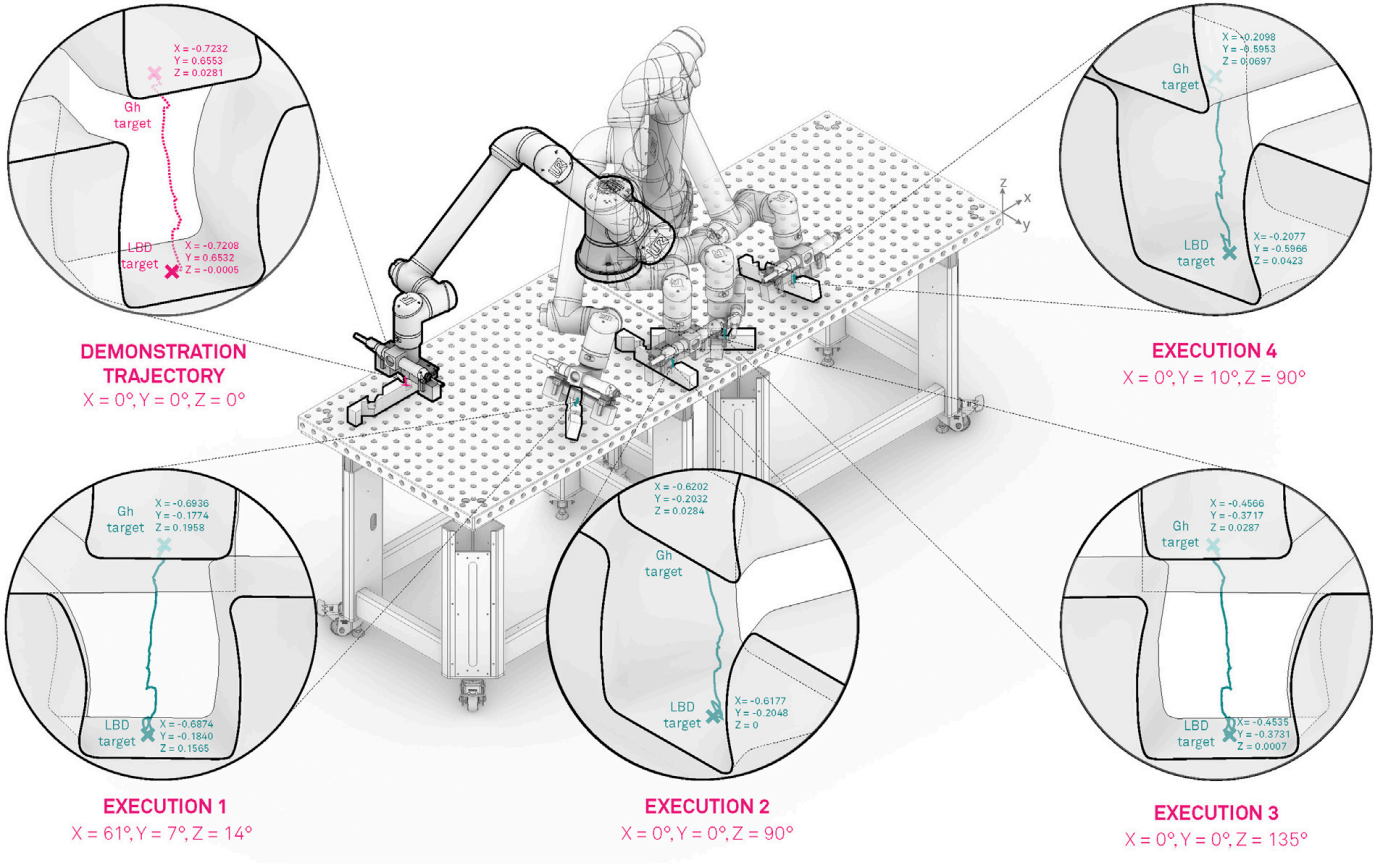
Modulation and execution of the demonstrated motion in various locations of the robot workspace.

**FIGURE 8:**
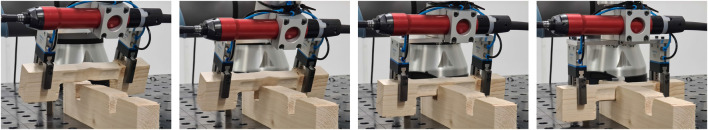
Execution of the demonstrated trajectory in 4 phases (approach **(left)**, alignment **(middle-left)**, interlocking **(middle-right)** and final configuration **(right)**).

**FIGURE 9:**
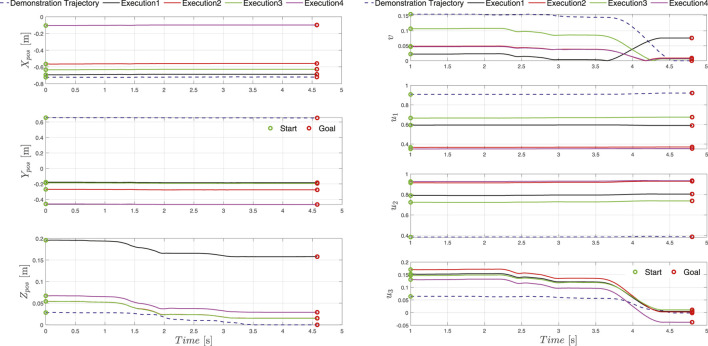
Execution position **(left)** and orientation **(right)** trajectories recorded at five locations in the workspace of the robot. The goal location given by the global design unit is depicted as a red circle, whereas the transformed start location of the trajectory is presented with a green circle. The demonstrated trajectory is depicted with dashed lines and the transformed executed trajectories are presented with solid lines.

**FIGURE 10:**
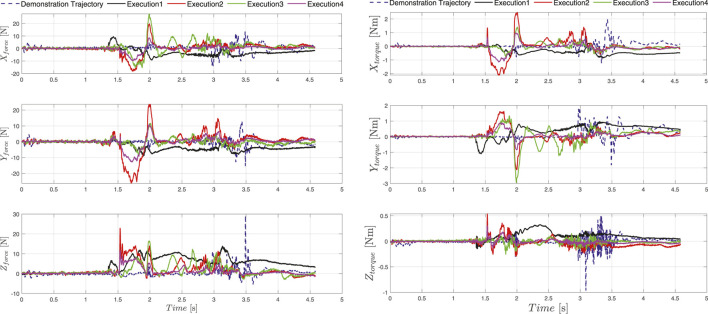
The demonstrated force-torque profile is represented with dashed blue line, solid lines represent forces and torques during the execution of the demonstrated assembly tasks at different locations of the timber studs in the workspace of the robot.

In the initial phase we recorded several demonstrations of the given joint assembly task. Initially the human operator set the robot into free drive mode e.g., active gravity compensation, with the help of the HRI interface and started to demonstrate the desired movement. Guiding the UR10e robot is substantially harder then compared to a KUKA iiwa or Franka panda robot, which come with active torque measurement in each joint, making the gravity compensation mode feel much smoother in comparison whit the UR10e, where the joint torques are estimated threw motor current measurement. In our previous work (Kramberger et al. (2017)) we applied iterative learning control coupled with force adaptation in order to optimize the demonstrated trajectory providing good results compared to an untrained human demonstrator. In this work we skipped this step and trained the human operator to perform the assembly task as best as possible with the robot. The outcome was recorded and is visualized in Figure 7 in the upper left zoomed in field with pink. A visual representation of the demonstration phases is given in Figure 8 and the time sequence of the recorded robot positions in Figure 9 with blue dashed lines. After the demonstration phase, we re-executed the kinematic trajectory and recorded the net force-torques arising during the demonstration which can be seen in Figure 10 represented with dashed lines. The force-torque measurement serves as a ground truth data for later comparison with the compliance execution, which will be elaborated in the following section.

For proving the effectiveness of the proposed framework, we manufactured pine timber blocks with a KUKA KR240 R3330 industrial robot which is quipped with a CNC end mill and the KUKA CNC software package. The robot toolpath was defined in the Computer Aided Manufacturing (CAM) extension of the Fusion 360^2^ software. Afterwards, the generated G-code was parametrically translated into robot movements using the KUKA PRC (Braumann and Brell-Cokcan, 2011) plug-in for Grasshopper. With this approach, a 0.5 mm fabrication tolerance was achieved and thus we can ensure a tight connection and at the same time provide enough space for the execution of the demonstrated assembly motion. Furthermore, the manufacturing process left uneven surface finish on the test studs, which are the outcome of the imperfections in the wood. For this reason, the manufactured studs slightly differ one from another and therefore, to ensure a successful robotic assembly, the force adaptation procedures have to be applied.

### 6.2 Execution of the Demonstrated Assembly Task in Connection With the Digital Twin

After the demonstration phase, the obtained data was stored in the database associated with the assembly digital twin. In the execution digital twin the CDMP execution is defined as a service call, with the input corresponding to the new pose goal of the demonstrated trajectory, specified by the global design unit. The output of the service is a positional and dynamical signal, characterizing the success rate of the execution. The success rate is evaluated based on:

• comparing the new goal pose given by the global design unit and the actual measured Tool Center Point (TCP) pose of the robot after the execution finished. If the poses match, the execution was successful, on the other hand it signals a failure and the execution is repeated.
• Force-torque measurements during the execution. If the forces and torques, exceed a threshold defined by the average force and torque value from the net forces and torques recorded during the replay of the demonstration, the execution fails and vice versa.

The evaluation mechanism, signals the assembly digital twin to proceed with the assembly plan, re-execute the assembly sequence or set the robot into collaboration mode signalling the human to assist with the assembly. Similar evaluation procedures were implemented in our previous work dealing with collaborative robot screwing (Naboni et al. (2021)) and were extended in this work to accommodate the assembly execution with demonstrated data.

In total we evaluated 100 executions randomly covering the entire workspace of the robot with a success rate of 93%. The assembly execution sequence segmentation in 4 phases e.g. approach (new transformed start configuration), alignment, interlocking and final configuration (new goal pose specified by the global design unit) is depicted in Figure 8. Furthermore, the five execution examples where the orientation and position is significantly changed compared to the demonstrated trajectory are shown in Figures 7–9 for the kinematic trajectories and in Figure 10 for the dynamic trajectory. In the seven failed execution cases, the evaluation mechanism was triggered mainly based on too high execution forces. This was the outcome of imprecision’s in the placement of the assembly strut on the table, misalignment discrepancy during the grasping of the assembled strut or manufacturing imperfections which caused jamming at certain test locations. To mitigate this problem the goal pose given by the global design unit was re-evaluated and aligned by hand so that it matched, and in the second try the assembly strut was re-grasped from the fixture and the assembly re-executed. Focusing on the execution of the assembly task, it can be seed in Figure 10 that the execution force-torque measurements (solid lines) are on average lower then the force-torque measurements recorded after the demonstration (dashed lines). The application of the force-torque adaptation method, described in Sec. 5.4, contributed to a better overall performance of the execution.

Assembly of wooden trusses can in almost all cases be considered as a planar assembly problem, therefore the variations in assembly joint orientation is considered around a single axis e.g., robot TCP defined *z-*axis. For this reason, we conducted the majority of the experiments on the *x-y* plane (table plane), two instances can be observed in Figures 7–9 outlined as Execution two and 3. To show that the orientation transformation method described in Sec. 5.3.1 works for all arbitrary given orientations, we conducted an experiment with a target orientation specified in *x-y* and *y-z* plane (Execution 4) and a target orientation and position specified in all six principal axis (Execution 1). The successful execution of the assembly task is visible in Figures 7–9 and in Figure 10 with black and magenta color, showing that the force-torque measurement during the execution is comparable with the planar executions, furthermore proving that the accuracy of the proposed transformation method works adequately.

## 7 Conclusion

In this paper we propose a framework for assembly of complex timber trusses with a robotic manipulator. In the robotics domain the task can be related to the peg in hole assembly problem, where small positional discrepancies, usually caused by imprecise grasping, positioning of the work peace or the manufacturing process, lead to high forces applied on the work peace or manipulator. Furthermore, the assembly task cannot be programmed in an efficient way with conventional robot programming techniques. Therefore, we utilize LbD to transfer the human assembly skills onto the robotic system. For this reason we devised a teaching robot cell, where the human expert operator can teach the relevant assembly motions, which can later be transferred to the execution cell. In the paper we introduce the concept of the assembly digital twin, which directly links the architectural truss and structure design, with the robotic assembly, which is further enhanced with assembly methods utilizing learning by demonstration. With this setup we can efficiently and with a high success rate, assemble timber structures in layered fashion as well as complex joinery.

The aim of this work is to show the synergy between the structural design process and robot execution, with witch we are able to perform complex assembly tasks utilizing LbD and execute them at any given position and angle of the timber truss given by the global structural design. Therefore, we harnessed the modulation properties of the CDMPs and enhanced them with a novel orientation transformation system, which enables us to precisely transform the entire encoded motion according to the pose specified by the global assembly designer. Furthermore, we coupled a force compliance controller directly to the acceleration part of the CDMP, to facilitate adaptation to environmental changes during the assembly execution, thus minimizing the interaction force-torques and maximizing the success rate of the assembly.

The performance of the proposed framework was evaluated in several experiments, where a single human assembly demonstration was executed under various poses in the workspace of the robot. The pose input was provided by the global designer and used for transforming the demonstrated trajectories. We demonstrated, that a complex joint can be assembled at any arbitrary pose in the robots workspace, with a high success rate, despite the manufacturing imperfection in the wooden studs. The experiments also show, that the added compliance in the system adds to a higher success probability, and efficiently compensates for misalignment’s and manufacturing errors of the designed wooden studs, which in turn increases the robustness of the assembly strategy.

In future work, we will focus on extending the framework in order to be integrated with the overall timber assembly system, we are building. Furthermore we will investigate the possibility of up scaling, in order to facilitate even more complex and big timber trusses with a larger robot.

## Data Availability

Enquiries related to the data presented in the original contribution, should be addressed to the corresponding author directly.

## Footnotes

1
https://www.rhino3d.com/.

2
https://www.autodesk.com/products/fusion-360/overview
